# IDH1-Associated Primary Glioblastoma in Young Adults Displays Differential Patterns of Tumour and Vascular Morphology

**DOI:** 10.1371/journal.pone.0056328

**Published:** 2013-02-22

**Authors:** Sergey Popov, Alexa Jury, Ross Laxton, Lawrence Doey, Naga Kandasamy, Safa Al-Sarraj, Juliane M. Jürgensmeier, Chris Jones

**Affiliations:** 1 Division of Molecular Pathology, The Institute of Cancer Research, Sutton, United Kingdom; 2 Division of Cancer Therapeutics, The Institute of Cancer Research, Sutton, United Kingdom; 3 Department of Neuropathology, King’s College Hospital, London, United Kingdom; 4 Department of Neuroradiology, King’s College Hospital, London, United Kingdom; 5 AstraZeneca, Alderley Park, Macclesfield, United Kingdom; Robert Wood Johnson Medical School, United States of America

## Abstract

Glioblastoma is a highly aggressive tumour with marked heterogeneity at the morphological level in both the tumour cells and the associated highly prominent vasculature. As we begin to develop an increased biological insight into the underlying processes driving the disease, fewer attempts have thus far been made to understand these phenotypic differences. We sought to address this by carefully assessing the morphological characteristics of both the tumour cells and the associated vasculature, relating these observations to the *IDH1*/*MGMT* status, with a particular focus on the early onset population of young adults who develop primary glioblastoma. 276 primary glioblastoma specimens were classified into their predominant cell morphological type (fibrillary, gemistocytic, giant cell, small cell, oligodendroglial, sarcomatous), and assessed for specific tumour (cellularity, necrosis, palisades) and vascular features (glomeruloid structures, arcades, pericyte proliferation). *IDH1* positive glioblastomas were associated with a younger age at diagnosis, better clinical outcome, prominent oligodendroglial and small cell tumour cell morphology, pallisading necrosis and glomeruloid vascular proliferation in the absence of arcade-like structures. These features widen the phenotype of *IDH1* mutation-positive primary glioblastoma in young adults and provide correlative evidence for a functional role of mutant *IDH1* in the differential nature of neo-angiogenesis in different subtypes of glioblastoma.

## Introduction

Glioblastoma is a highly vascularised tumour, with the WHO diagnostic criteria recognising the importance of microvascular proliferation in the differential diagnosis with lower grade astrocytic tumours, the latter having markedly better prognosis [1]. These lower grade lesions frequently progress according to the so-called clinical secondary glioblastoma pathway to grade IV lesions, and are strongly associated with mutations in the isocitrate dehydrogenase genes (*IDH*1, and rarely, *IDH2*), lower age of onset, and better prognosis [2].

Less frequently, clinical *de novo* or primary glioblastoma may also be associated with young adults and *IDH1* mutation [3], by which time the extensive microvascular proliferation associated with the higher grade tumours is already present. As with other phenotypic and genotypic elements of glioblastoma development, the specific patterns of vascular proliferation in glioblastoma may also exhibit a wide range of heterogeneity, although clinicopathological and molecular correlates of these observations are ill-defined.

Angiogenesis may be mediated by VEGF, an endogenous cytokine that stimulates capillary sprouting from pre-existing vessels towards VEGF-expressing tumour cells. Tumour VEGF expression and angiogenesis are mainly hypoxia-driven but can also be promoted by other vascular cytokines and may be constitutively activated as a result of mutation [4]. *IDH1* mutation results in the formation of the oncometabolite 2-hydroxyglutarate and the induction of the hypoxia-inducible factor subunit HIF-1α, a transcription factor that facilitates tumour growth and angiogenesis in low oxygen conditions [5].

IDH1 mutant glioblastomas have been shown to differ in their epigenetic and genomic presentation [6], [7]. A new model has been proposed in which the phenotypic features which distinguish IDH1 mutant tumours are those associated with lower grade lesions, supporting their development as part of a progression pathway regardless of clinical presentation [8]. Differences in demographics and tumour location between IDH1 mutant and wild-type glioblastomas further suggest different aetiologies of what may be seen to be two distinct disease entities [8].

Treatment strategies aimed at minimising the extent of neovascularisation are being extensively studied in glioblastoma patients in combination with the standard of care ‘Stupp’ protocol [9] of chemoradiotherapy with temozolomide, as well as with novel molecularly targeted agents [10], [11]. In this context, promoter methylation of the DNA repair enzyme O^6^-methylguanine-DNA-methyl-transferase (MGMT) provides important predictive power, with survival benefits largely restricted to the subset of patients lacking protein expression [12].

With the increasing understanding of the underlying biology of primary glioblastoma, concurrent assessment of the inherent phenotypic heterogeneity of the disease has been lacking. We have sought to address this by carefully assessing the morphological characteristics of both the tumour cells and the associated vasculature, and relating these observations to the *IDH1*/*MGMT* status, with a particular focus on the early onset population of young adults who develop primary glioblastoma. We identify specific subtypes of tumour and vascular biology which are closely linked with IDH1 mutation in young adults.

## Materials and Methods

### Patient Samples

We retrieved after approval from Wandsworth Research Ethics Committee a series of 276 samples from patients diagnosed with glioblastoma (WHO grade IV) from the archives of King’s College Hospital, London, with histopathological diagnosis (SP, SA-S) and radiographic features (SP, NK) confirmed by re-review. All the samples enrolled in the present study were unlinked and unidentified from their donors. Due the retrospective nature of the study, no written informed consent from patients was obtained, with the exception of the UK samples obtained after 2006, where all patients signed a written informed consent, following the UK Human Tissue Act approved in that year. The age range of the patients was from 26–83 years, with a median of 58 years, and comprised 61% males to 39% females. Clinical follow-up was available for 263 patients, with a median survival of 5.7 months (range 4 days –5.6 years). A full description of all data relating to these samples in this study is given in Supplementary Table S1.

### Tissue Microarray Construction

Up to four 1 mm diameter cores were taken per block, and constructed in four TMAs containing approximately 90–100 samples each. In addition, a series of control tissues were included, comprising normal human brain, normal mouse brain, placenta, colon and lung. A further H&E was taken of each TMA and re-reviewed to ensure the presence of tumour cells.

### Histopathology

A detailed morphological assessment of the GBM samples was also carried out and the predominant tumour subtype (where more than 20% of the cells are present with this appearance) - fibrillary, oligodendroglial, sarcomatous, gemistocytic, small cell or giant cell - recorded. Discrepancies between reviewers (n = 24) were assigned by consensus. We compared the density of the vascular network and the presence of vascular changes (glomeruloid structures, endothelial and pericyte proliferation, arcade/garland like vascular structures) in the most cellular part of the tumour, around palisade structures and large necrotic areas, at the tumour-normal brain border, and in the normal brain.

Glomeruloid structures show several closely packed small vessels running in parallel fashion, which in cross-sections is seen as a tufted collection of vessels reminiscent of kidney glomeruli. Arcade-like vascular proliferations are seen as semicircular garlands of hypercellular, often glomeruloid vessels around regions of necrosis. Assessment of pericytes was facilitated by morphological appearance and negativity for CD34, CD105, and GFAP.

### Immunohistochemistry

Each TMA was serially sectioned at 5 µm and immunohistochemistry was performed. Slides were dewaxed in xylene and rehydrated through a descending ethanol series. The mouse EnVision™+ HRP System, (DAKO, Carpinteria, CA, USA) was used for GFAP (MO761) (DAKO), Vimentin (M0725) (DAKO), and IDH1^R132H^ (DIA H09) (Dianova, Hamburg, Germany). Antigen retrieval was performed by boiling the slides for 20 min in 10 mM citrate buffer pH6 in a coplin jar and cooled on the bench for 20 min. Each section was blocked with DAKO Protein block (X0909) for 10 min at room temp and incubated with primary antibody for 1 hour at room temp (GFAP 1∶50, Vimentin 1∶100, IDH1^R132H^ 1∶20). The Universal R.T.U. Vectastain Elite ABC Kit (Vector Laboratories, Burlingame, CA, USA) was used for CD105 (4G11) (Leica Microsystems, Newcastle, United Kingdom) and CD31 (JC70A) (DAKO). Antigen retrieval was performed by boiling the slides at pressure for 2 min in 10 mM citrate buffer pH6. After blocking, each section was incubated with primary antibody for 1 hour at room temp (CD105 1:100, CD31 1:30). Staining was completed with a 5 minute incubation with DAKO 3,3′-diaminobenzidine (DAB)+ substrate-chromogen and counterstained with Mayer’s Haematoxylin (Sigma-Aldrich, Poole, UK).

### Genetic Analysis

DNA was extracted from paraffin-embedded formalin-fixed tissue using the QIAamp DNA Micro Kit (Qiagen, Crawley, UK). Primers for the 129 bp *IDH1* fragment were designed and PCR carried out according to the method in Hartmann *et al.* [13]. The PCR product was sequenced and the *IDH1^R132H^* mutation analysed using Mutation Surveyor (SoftGenetics, Pennsylvania, USA) and manually with 4Peaks (Mekentosj, Aalsmeer, The Netherlands). *MGMT* promoter methylation was assessed by bisulfite conversion of the FFPE-extracted DNA, performed using the Qiagen EpiTect Bisulfite Kit and Methylation-Specific PCR (MSP).

### Statistical Analysis

All statistical tests were performed in R2.11.0. Correlations between categorical variables were analysed by Fishers exact test. Cumulative survival probabilities were calculated using the Kaplan-Meier method with differences between survival rates analysed with the log-rank test. Important prognostic information (including Karnofsky performance score) was not available for all cases in this retrospective study, so multivariate analysis was not able to be performed. All tests were two-tailed, with a confidence interval of 95%. P values of less than 0.05 were considered statistically significant.

## Results

### Distinct Morphological Subtypes of Glioblastoma Cells

**Figure 1:**
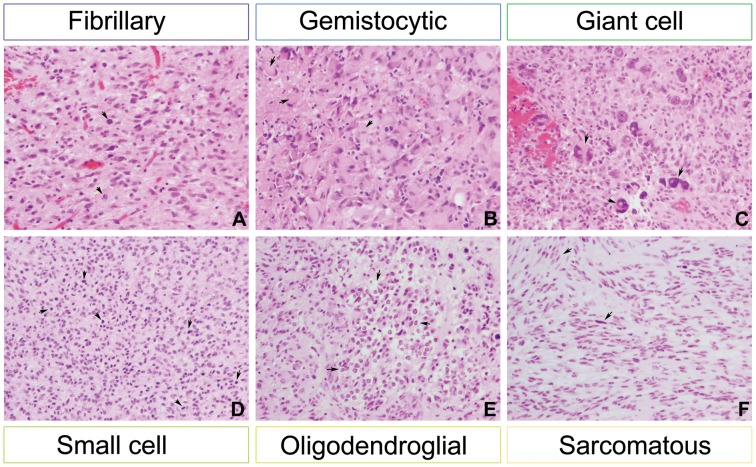
Distinct cell morphological types in clinical glioblastoma specimens. Examples of tumours are shown with predominant morphology classified as fibrillary (A, RMH5690, pleomorphic cells with pink cytoplasm and mitotic figures (arrows)), gemistocytic (B, RMH6716, cells with abundant glassy cytoplasm and eccentric nuclei (arrows)), giant cell (C, RMH6004, very large pleomorphic cells with hyperchromatic nuclei (arrows)), small cell (D, RMH5723, small cells with brisk mitotic activity (arrows)), oligodendroglial (E, RMH5970, ‘fried egg’ appearance of tumour cells (arrows)), and sarcomatous (F, RMH5966, disorganised fascicles of spindle cells (arrows)). All images original magnification×200, haematoxylin and eosin.

Glioblastomas are known for their marked histological heterogeneity, both across multiple cases but also with the cells of an individual’s tumour. We sought to quantify this by determining the predominant cell type present in tumours from our cohort. Tumours were classified either as fibrillary (composed of pleomorphic cells with scant or more prominent pink cytoplasm; some cells can demonstrate cytoplasmic extensions; Figure 1A), gemistocytic (abundant, glassy cytoplasm, eccentric nuclei; Figure1B), giant cell (composed primarily of bizarre, pleomorphic, often multinucleated cells; Figure 1C), small cell (monomorphic round to oval nuclei, uniform in size with bland chromatin but with high mitotic activity; occasional clear perinuclear haloes, branching capillaries and microcalcifications can be seen; Figure1D), oligodendroglial (cytologic monotony with rounded, bland nuclei surrounded by perinuclear haloes; in some cases branching, “chicken wire”-like capillaries; Figure 1E) or sarcomatous (consisting of fascicles of spindle cells, sometimes with collagen depositions; stellate cells in a myxoid stroma can be seen in some cases; Figure 1F).

**Figure 2:**
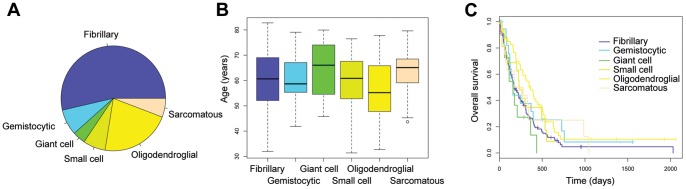
Distribution and clinical significance of predominant cell morphology in glioblastoma. (A) Pie chart showing proportion of tumours with predominant fibrillary (purple, 53.6%), gemistocytic (blue), 7.9%, giant cell (green, 3.9%), small cell (light green, 6.9%), oligodendroglial (yellow, 21.7%) and sarcomatous (orange, 5.8%) morphology. (B) Boxplot showing the age distribution of the morphological subtypes (C) Kaplan-Meier curve showing association of predominant cell morphology and clinical outcome (overall survival).

The predominant tumour subtype was fibrillary in 148/276 (53.6%) cases, gemistocytic in 22/276 (7.9%), giant cell 11/276 (3.9%), small cell 19/276 (6.9%), oligodendroglial 60/276 (21.7%) and sarcomatous 16/276 (5.8%) (Figure 2A). There was a trend towards prognostic significance of cellular morphology with respect to overall survival (p = 0.0632, log-rank test) (Figure 2B). There was driven by a significantly better survival associated with oligodendroglial differentiation *versus* the remaining tumours (p = 0.00729, log-rank test), and a significantly worse survival for those tumours with a predominance of giant cells (p = 0.0125, log-rank test) (Figure 2C).

Oligodendroglial and small cell morphologies were significantly over-represented in the younger age group (13/60, 22% vs 9%, p = 0.006; 4/19, 21% vs 28/257, p = 0.039, Fishers exact test) as well as those tumours with *IDH1* mutation (3/59, 5% vs 5/215, 2%, p = 0.08; 2/19, 11%, 6/255, 2%, p = 0.04, Fishers exact test).

There was a significantly reduced frequency of MGMT promoter methylation in gemistocytic tumours (2/15, 13% vs 91/232, 26%, p = 0.03, Fishers exact test), and a significantly greater frequency in oligodendroglial (32/59, 54% vs 61/188, 32%, p = 0.001, Fishers exact test) and giant cell (7/11, 64% vs 86/236, 36%, p = 0.05, Fishers exact test), despite the different prognoses of these latter two morphological subgroups. There was no significant difference in MGMT promoter hypermethylation in the younger age group (5/11, 45% vs 88/236, 37%, p = 0.21, Fishers exact test). A summary of clinicopathological and molecular correlates of differing glioblastoma morphologies are given in Table 1.

**Table 1:** Clinicopathological correlates of predominant cell morphology in glioblastoma.

	Age (years)	Survival (months)	IDH1 mutation	MGMT methylation
*All glioblastoma*	*60.0 y*	*5.7 m*	*8/274 (2.9%)*	*93/247 (37.7%)*
Fibrillary	60.7 y	4.9 m	2/147 (1.4%)	38/131 (29.0%)
Gemistocytic	58.7 y	4.1 m	0/22 (0%)	2/15 (13.3%)
Giant cell	66.1 y	5.3 m	1/11 (9.1%)	7/11 (63.6%)
Small cell	60.9 y	7.3 m	2/19 (10.5%)	9/18 (50.0%)
Oligodendroglial	55.2 y	8.9 m	3/59 (5.1%)	32/59 (54.2%)
Sarcomatous	65.1 y	7.5 m	0/16 (0%)	5/14 (35.7%)

### Patterns of Vascularisation in Glioblastoma

**Figure 3:**
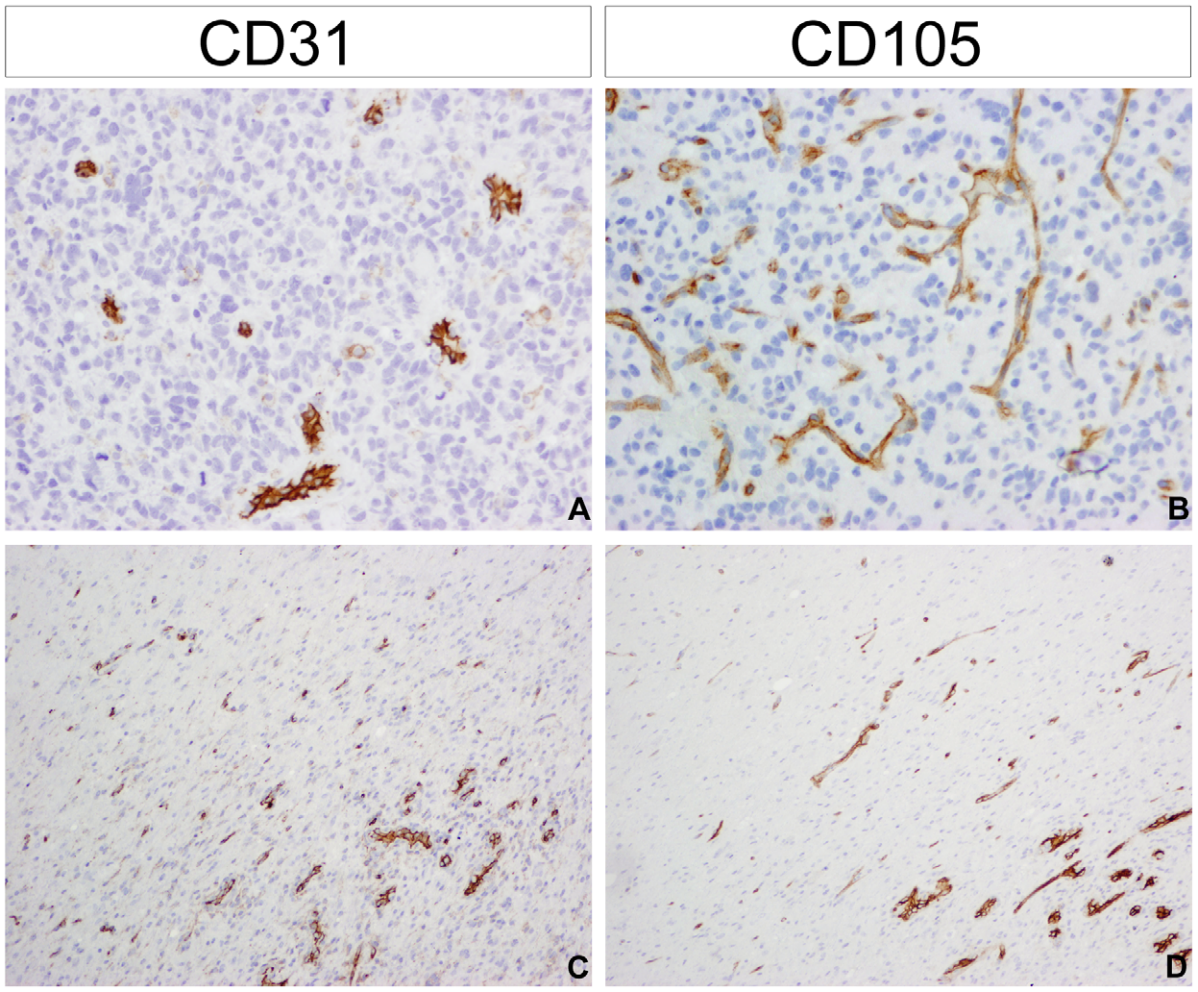
Patterns of vascularisation in glioblastoma. (A) Moderate increase of normal-looking or angulated/saw-like vessels positive for CD31. RMH5685 (B) Highly branching vessels positive for CD105. RMH5714 (C) CD31 staining and (D) CD105 staining highlighting increased vascular density and prominent microvascular proliferation in the transitional area between the highly infiltrating tumour edge and normal brain tissue. RMH6950 All images original magnification×100.

Different areas showed a variable vascular pattern. Highly cellular areas of the tumour showed low vascularisation or a generalised moderate increase of normal-looking or angulated/saw-like vessels with prominent CD31 staining (Figure 3A). There were occurrences of highly-branching vascular architecture with strong CD105 staining (Figure 3B) and in rare cases vasculogenic mimicry was noticed. It was also noticed that the level of vascularisation in normal brain adjacent to the tumour usually demonstrated an increased vascularisation, with the transitional area between the highly infiltrating tumour edge and normal brain tissue itself frequently associated with an increased microvessel density (Figure 3C,D). A prominent microvascular proliferation was observed rarely, and only in cases with necrotic areas located close to the normal brain/tumour border.

**Figure 4:**
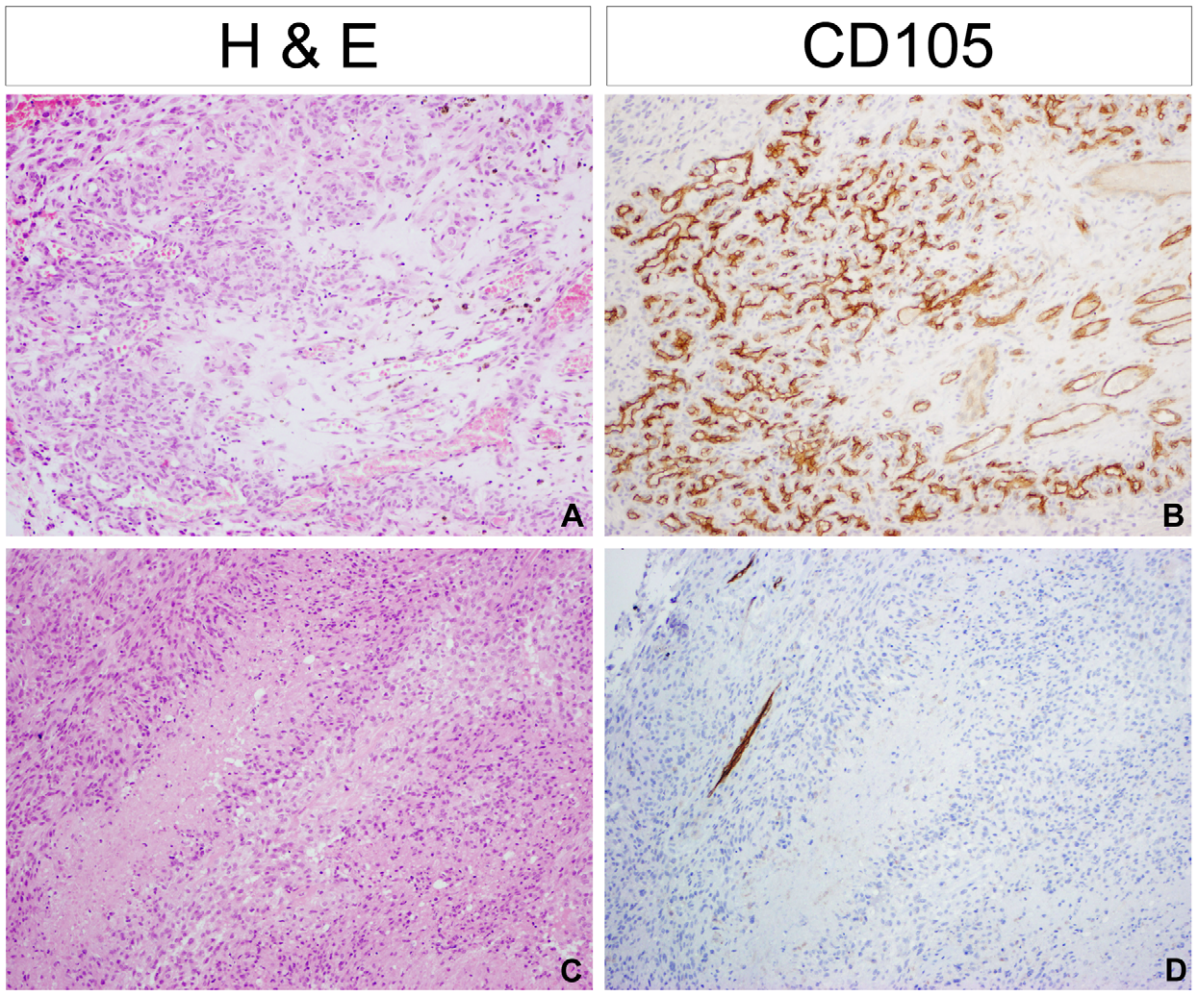
Differential patterns of vascularisation and necrosis. (A) Prominent vascularisation occurs around large necrotic areas of the tumour; (B) High expression of CD105 reflects active neovascularisation in these areas. RMH5967. (C) Regions with prominent pallisading necrosis were poorly vascularised; (D) Only scattered CD105-positive vessels were often observed. RMH5725 All images original magnification×100.

The most prominent vascularisation occurs around large necrotic areas of the tumour (Figure 4A), with high expression of CD105 reflecting active neovascularisation in these areas (Figure 4B). This was in distinct contrast to regions in which there was prominent pallisading necrosis, which were found to be not well, and even poorly vascularised (Figure 4C). Here there were often only scattered vessels with no greater than moderate staining for CD31 and CD105 (Figure 4D). Areas of pallisading necrosis were significantly associated with oligodendroglial (38/60, 63% vs 118/216, 55%, p = 0.05, Fishers exact test) and small cell (15/19, 79% vs 141/257, 55%, p = 0.024, Fishers exact test) morphologies, as well as the younger age group (<40 yrs) (10/12, 83% vs 146/264, 55%, p = 0.04, Fishers exact test) and *IDH1* mutation (7/8, 88% vs 149/266, 56%, p = 0.05, Fishers exact test). There was no association with MGMT promoter methylation.

**Figure 5:**
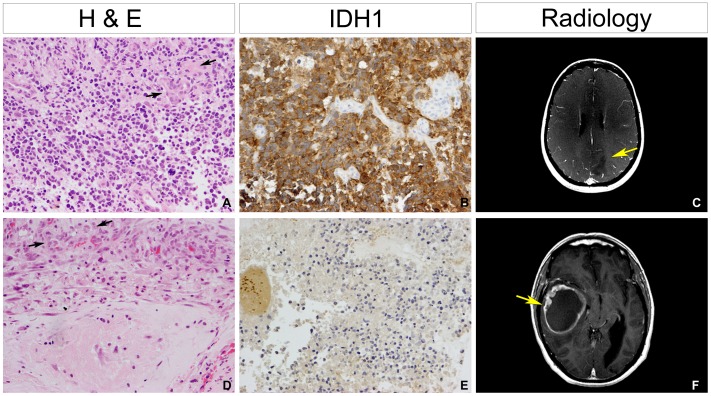
Association of specific vascular architecture with IDH1 status and young age. (A) Example of glomeruloid appearance of tumour vessels (arrows). Haematoxylin and eosin. (B) Tumour cell positivity for mutant IDH1 associated with glomeruloid vessels. RMH5984. (C) Contrast-enhanced T1-weighted images of patient RMH5984, showing an incompletely enhancing lesion (arrow). (D) Tumour vessels forming arcade-like structures (arrows). Haematoxylin and eosin. (E) All tumours with arcade-like vascular structures were negative for IDH1 mutation by immunohistochemistry (and direct sequencing). RMH6642. (F) Contrast-enhanced T1-weighted images of patient RMH6642, with pronounced ring-like enhancement (arrow). All histopathological images original magnification×200.

Vessels tended to present in glomeruloid (several cross-sectioned vascular lumens with or without cell proliferation; 175/272) and arcade-like structures (semi-circular garlands of vessels, 48/272). We did not observe intraluminal endothelial proliferation in any cases. Tumours with glomeruloid structures (Figure 5A) were particularly associated with a predominantly small cell morphology (10/19, 53% vs 88/261, 34%, p = 0.04, Fishers exact test), *IDH1* mutation (7/8, 88% vs 139/262, 63%, p = 0.05, Fishers exact test) (Figure 5B), younger age at diagnosis (6/12, 50% vs 71/260, 27%, p = 0.06, Fishers exact test). These cases demonstrated an incomplete radiographic contrast enhancement by contrast-enhanced T1-weighted imaging (Figure 5C). By contrast, arcade-like structures (Figure 5D) were restricted to *IDH1* wild-type tumours (Figure 5E) (0/8 *IDH1* mutant vs 48/262, 18%) (Figure 5D), which were also never observed in patients under the age of 40 years at diagnosis (0/12 vs 48/260, 18%, p = 0.09, Fishers exact test). Such tumours showed complete, ‘ring-like’ contrast enhancement on contrast-enhanced T1-weighted images (Figure 5F).

Finally, the nature of the vascular proliferation was investigated in more detail, with close attention paid to the cells which comprised these structures. A hyperproliferation of pericytes was observed in 241/272 (88.6%) cases. This pericyte proliferation was observed in all (8/8) cases of *IDH1* mutant tumours. There was a significant association of pericyte proliferation with glomeruloid structures (173/241 positive, 0/31 negative, p<0.001, Fishers exact test). There were no associations of vascular pattern with MGMT status.

## Discussion

According to recently-proposed models of gliomagenesis, *IDH1* mutation initiates a series of genetic and epigenetic cascades which produces a relatively homogenous group of glioblastomas presenting with a distinct phenotype with relatively good prognosis compared with the heterogeneous presentation of more aggressive *IDH1* wild-type tumours [2], [8]. Indeed, *IDH1* mutation appears sufficient to produce the distinctive G-CIMP hypermethylator phenotype associated with a younger age of presentation and differential gene expression profile [14].

In the present study, we noted strong associations of glioblastoma in young adults to harbour *IDH*1 mutation, prominent oligodendroglial and small cell tumour cell morphology, and glomeruloid vascular proliferation. Whilst oligodendroglial differentiation had been noted in *IDH1* mutant tumours previously [15], the observation of a significant association with neoplastic small cells is novel. Although previous studies have implicated small cell histology with EGFR amplification in glioblastoma [16]–[18], certain morphological characteristics are found in common between glioblastomas with extensive oligodendroglial and small cell components including haloes, perineuronal satellitosis and microcalcifications [19].

A recent report [21] correlated large areas of necrosis to the Mesenchymal gene expression subtype reported by molecular profiling studies [3], [7]. In our series, we identified tumours with small cell and oligodendroglial features (hallmarks more closely linked to a Proneural subtype) to be strongly linked to areas of pallisading necrosis, which have recently been associated with response to chemoradiotherapy [20]. Pseudopalisades are considered severely hypoxic, overexpressing HIF-1α and secreting proangiogenic factors such as VEGF and IL-8 [21]. It has been suggested that pseudopalisades represent a wave of tumor cells actively migrating away from central hypoxia, the driving force for which may be the *IDH1* mutation found in association with this abnormality in the present study.

Prominent neovascularisation is thought of as a secondary event in glioblastoma development, driven by hypoxia and necrosis. *IDH1* mutation itself may not be sufficient to account for a general HIF-1α-driven hypoxia in glioblastoma, as elevated HIF-1α levels are usually confined to severely hypoxic areas of necrosis [22] such as palisades. In fact a recent support suggests that the oncometabolite formed as a result of IDH1 mutation, 2-hydroxyglutarate, leads to diminished levels of HIF-1α [23].

Our observations link young adults with *IDH1* mutation with specific morphological features of glioblastoma to these hypoxic, glomeruloid patterns of vascular proliferation. The specific nature of the glomeruloid vascular patterns in fact are probably better described as of ‘glomerulonephritis-type’ as they form globular masses that resemble the glomerular tufts of the kidney involved in an autoimmune process called glomerulonephritis [24]. Most cells in these structures are not endothelial but pericytes, as has been suggested by previous ultrastructural studies [25]. Anti-angiogenic therapies devised for these patients will need to take into account the extensive pericyte networks associated with these tumour vessels.

A striking observation was the strong negative association of this clinicopathological subgrouping with the more pronounced arcade-like vascular appearance that may be frequently observed in *IDH1* wild type tumours. This is entirely consistent with a recent study identifying *IDH1* mutant glioblastomas to contain a significant non-contrast-enhancing component [8], whereby it is these garlands of microvascular proliferation which underlie the specific enhancements of contrast agents frequently observed in glioblastoma patients. In our series too, the majority of *IDH1* mutant, arcade-negative tumours were non-enhancing, unlike *IDH1* wild type, arcade-containing tumours. Thus we offer evidence of the underlying cause of these distinct radiological findings.

Of note, the group of young adult, *IDH1* mutant tumours with these specific phenotypes may be functionally linked to a subset of paediatric glioblastomas driven by mutations in genes encoding the histone H3.3 variant [26]. Specific mutations in the *H3F3A* gene at or close to lysine residues marked by trimethylation appear to have profound implication for gene transcription, and it has been shown that 2-hydroxyglutarate may exert similar effects via histone methyltransferases [27]. A study investigating patterns of tumour cell morphology and vascular architecture in this context may provide valuable insights into these links.

## Supporting Information

Table S1 **Full clinicopathological and molecular data for all samples analysed.** Glioblastoma samples are listed along with outcome data, predominant cellular morphology and vascular features, as well as IDH1 and MGMT status.
(TXT)
